# The Intercellular Synchronization of Ca^2+^ Oscillations Evaluates Cx36-Dependent Coupling

**DOI:** 10.1371/journal.pone.0041535

**Published:** 2012-07-25

**Authors:** Sabine Bavamian, Helena Pontes, José Cancela, Anne Charollais, Sergei Startchik, Dimitri Van De Ville, Paolo Meda

**Affiliations:** 1 Department of Cell Physiology and Metabolism, University of Geneva School of Medicine, Geneva, Switzerland; 2 Bioimaging Core Facility, University of Geneva School of Medicine, Geneva, Switzerland; 3 Department of Radiology and Medical Informatics, University Hospital Geneva, Geneva, Switzerland; University of Bremen, Germany

## Abstract

Connexin36 (Cx36) plays an important role in insulin secretion by controlling the intercellular synchronization of Ca^2+^ transients induced during stimulation. The lack of drugs acting on Cx36 channels is a major limitation in further unraveling the molecular mechanism underlying this effect. To screen for such drugs, we have developed an assay allowing for a semi-automatic, fluorimetric quantification of Ca^2+^ transients in large populations of MIN6 cells. Here, we show that (1) compared to control cells, MIN6 cells with reduced Cx36 expression or function showed decreased synchrony of glucose-induced Ca^2+^ oscillations; (2) glibenclamide, a sulphonylurea which promotes Cx36 junctions and coupling, increased the number of synchronous MIN6 cells, whereas quinine, an antimalarial drug which inhibits Cx36-dependent coupling, decreased this proportion; (3) several drugs were identified that altered the intercellular Ca^2+^ synchronization, cell coupling and distribution of Cx36; (4) some of them also affected insulin content. The data indicate that the intercellular synchronization of Ca^2+^ oscillations provides a reliable and non-invasive measurement of Cx36-dependent coupling, which is useful to identify novel drugs affecting the function of *β*-cells, neurons, and neuron-related cells that express Cx36.

## Introduction

In vertebrate tissues, several mechanisms cross talk in a complex network to ensure intercellular coordination. A consistent feature of this network are the cell-to-cell channels made of connexins [1]. These transmembrane proteins belong to a multigene family of 20 proteins, which form hydrophilic cell-to-cell channels for the rapid intercellular exchange of cytosolic molecules [1]. Pancreatic *β*-cells, which secrete insulin and are central to the development of both type 1 and type 2 diabetes, express junctional channels made only of connexin36 (Cx36) [2]. Previous studies have demonstrated the contribution of this protein to several aspects of the *β*-cell function [2], [3], [4], [5]. Thus, transgenic mice and cell lines, that no more express Cx36 do not show synchronized transients of cytosolic Ca^2+^ ([Ca^2+^]_i_) when stimulated by natural secretagogues [4], [6]. As a result, cells lacking Cx36 do not release insulin in a normal pulsatile fashion, show an increased basal release of the hormone, and a decreased insulin output in response to glucose [4], [6], three alterations reminiscent of the defects observed in prediabetic states and type 2 diabetes [7], [8], [9], [10]. The *β*-cells of transgenic mice lacking Cx36 are also sensitized to the effects of streptozotocin, alloxan and cytokines that are implicated in the development of type 1 diabetes, whereas transgenic mice overexpressing this connexin are protected against the same conditions [11], [12]. These findings indicate that Cx36-dependent signaling plays a physiologically relevant role in the regulation of *β*-cell function/survival, which may be involved in the development and/or maintenance of the *β*-cell dysfunctions leading to diabetes.

Drugs targeting Cx36 may be useful to further elucidate the functions of this protein, as well as to improve *β*-cell function and survival in (pre)diabetic states. However, the screening of such drugs is complicated by the methods evaluating cell coupling, which are invasive and cumbersome [13] and, thus, poorly adapted to a high-throughput drug screening. To address this issue, we hypothesized that the evaluation of the cell-to-cell synchronization of secretagogue-induced Ca^2+^ transients, in an insulin-producing cell line mimicking the characteristics of primary *β*-cells, could represent a reliable, non invasive and sensitive approach to Cx36-dependent coupling. Here, we show that a method that evaluates a “synchrony index”, reflecting the average degree of intercellular synchronization of Ca^2+^ transients within large cell populations, indirectly reflects the extent of cell-to-cell coupling due to Cx36 channels. Using this method, several candidate molecules targeting Cx36 channels were identified, of which some interfered with selected aspects of *β*-cell function.

## Results

### Cx36-dependent Coupling of MIN6 Cells Impacts on the Intercellular Synchronization of Ca^2+^ Transients

**Figure 1:**
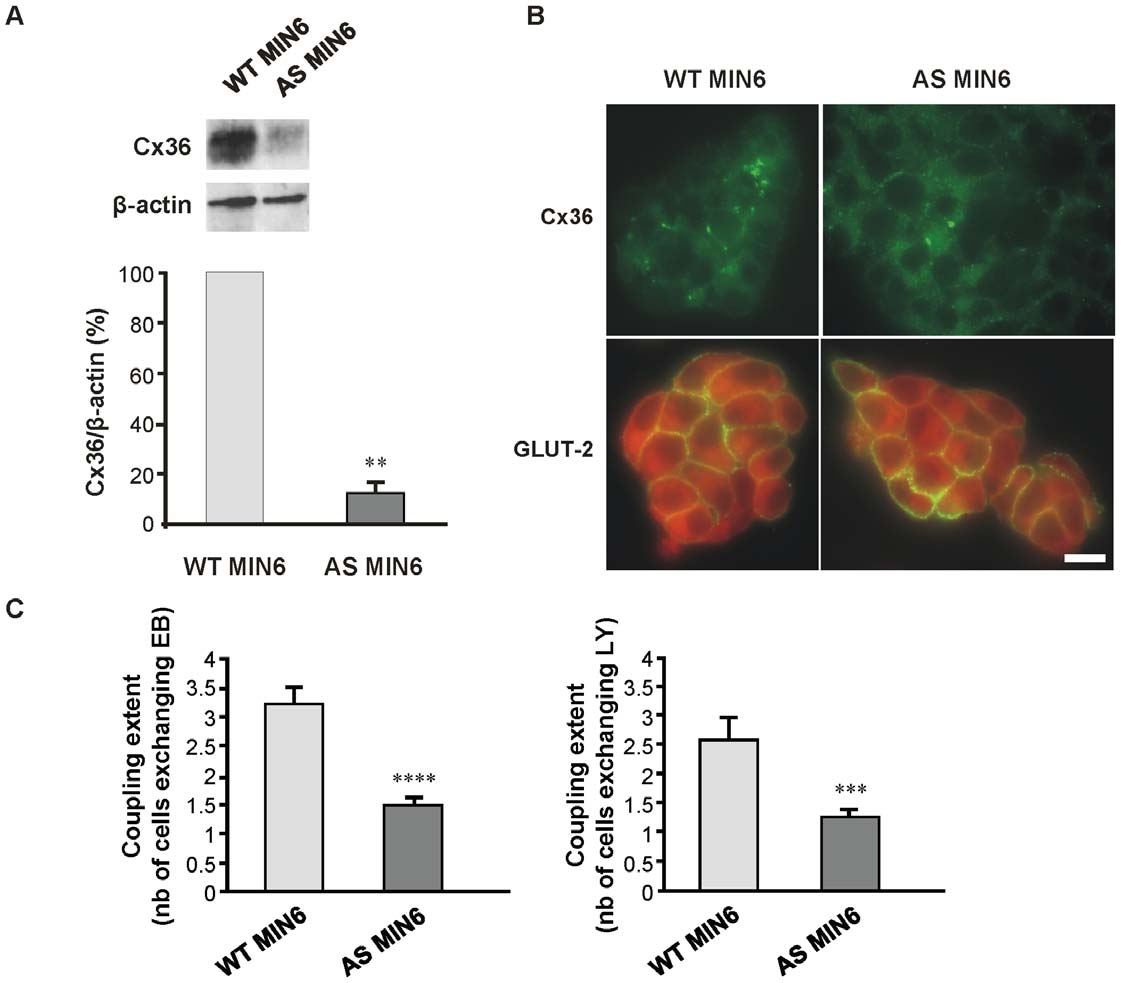
Wild type and Cx36-antisense MIN6 cells differ by the expression of Cx36 and cell-to-cell coupling. (*A*) Immunoblots of total cell extracts showed that AS Cx36 (*gray bar*) in comparison to WT MIN6 cells (*light gray bar*) featured a 80% reduction in Cx36 levels, relative to those of *β* smooth muscle actin. Data are means + SE of three independent experiments. **p<0.01; (*B*) Immunostaining showed that the expression Cx36, but not of GLUT-2, was lower in AS Cx36 than in WT MIN6 cells. Bar, 10 µm; (*C*) Microinjection of either EB or LY showed that AS MIN6 (*gray bar*) were significantly less coupled than WT cells (*light gray bar*). Values are means + SE of four independent experiments. ***p<0.001. The global evaluation of coupling, provided by computing a total coupling index, showed that the coupling of AS MIN6 was ∼3% of that of WT cells.

**Figure 2:**
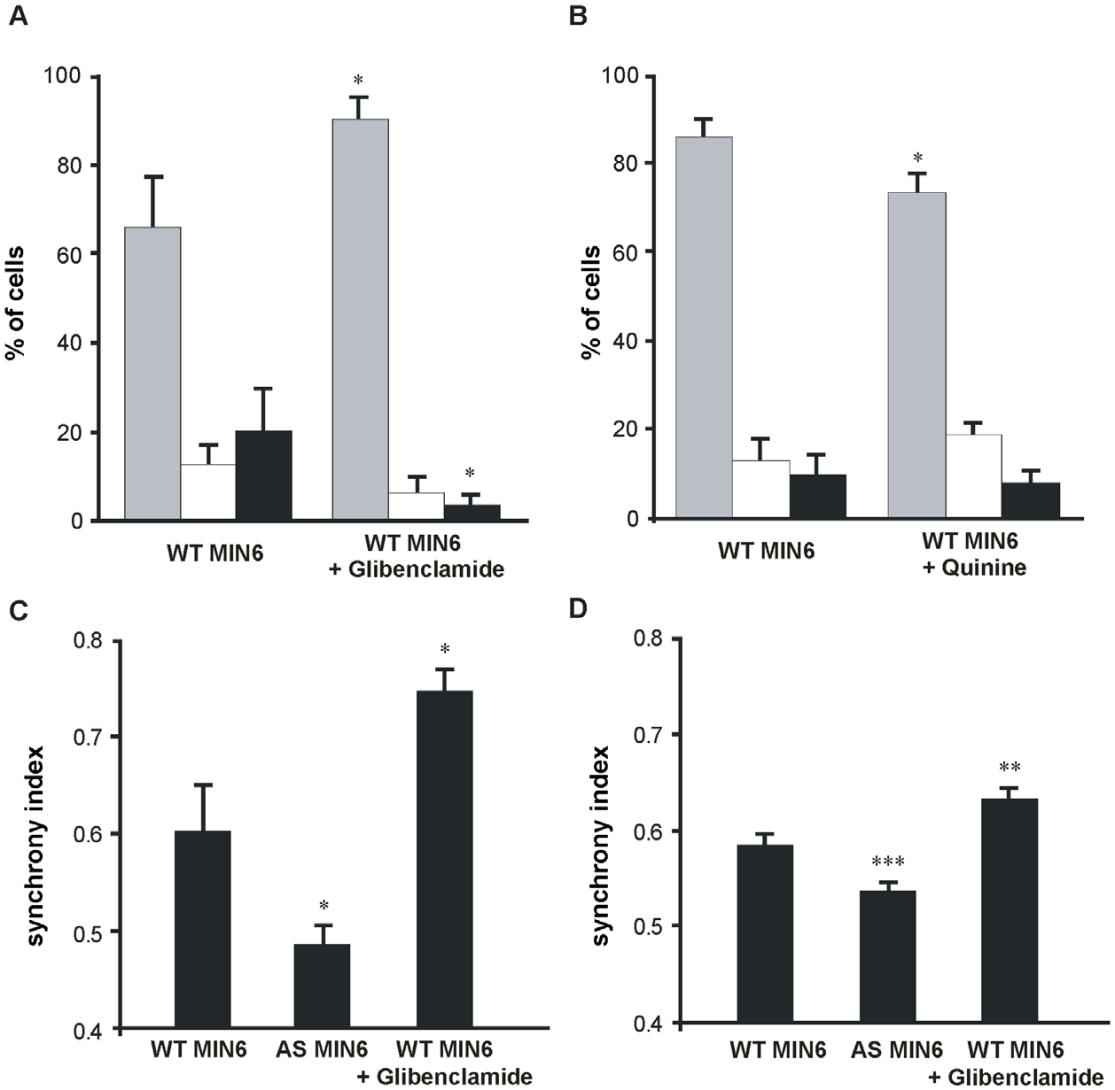
Glibenclamide and quinine differentially modulate the intercellular synchronization of glucose-induced Ca^2+^ oscillations. (*A*) During stimulation by 20 mM glucose in the presence of 15 mM TEA, MIN6 cells exposed for 24 h to 10 µM glibenclamide showed increased proportion of synchronous cells (*gray bars*) compared to control, but decreased proportions of asynchronous (*white bars*) and silent cells (*black bars*); (*B*) Under the same conditions, MIN6 cells exposed for 24 h to 10 µM quinine, showed a reduced proportion of synchronous cells (*gray bars*). Bars show means + SE values of three independent experiments. *p<0.05 versus values of WT MIN6 cells; (*C*) The synchrony index of WT MIN6 cells, as evaluated using a NIPKOW confocal microscope, was about 0.6. This value was decreased in AS MIN6 cells, and increased after exposure of the cells to glibenclamide; (*D*) Similar values of synchrony index data were semi-automatically calculated, in the same cell types, using an ImageXpress microscope. Data are means + SE of seven clusters measured for each condition in C, and 87 clusters measured for each condition in D. *p<0.05, **p<0.01 and ***p<0.001 versus values of WT MIN6 cells.

MIN6 cells natively express Cx36, at the exclusion of other connexin isoforms, and feature a stimulus-secretion coupling similar to that of primary *β*-cells [4], [6]. By stable transfection of an antisense mCx36-cDNA, we generated a clone of MIN6 cells (AS MIN6) which expresses significantly reduced levels (∼20% of controls) of Cx36 protein (Fig. 1 *A* and *B*), and normal distribution of the GLUT-2, a transporter essential for glucose uptake during the insulin secretion process (Fig. 1 *B*). Using standard confocal microscopy, we observed that, in contrast to wild type controls, which show synchronous Ca^2+^ oscillations when stimulated by 20 mM glucose and 15 mM TEA, most AS MIN6 cells featured either asynchronous or nil Ca^2+^ oscillations (Fig. S1). Microinjection of both LY and EB further documented that the coupling of AS MIN6 cells was significantly reduced compared to that of wild type controls (Fig. 1 *C*, Table 1). Exposure of wild type MIN6 cells (WT MIN6) to glibenclamide, a sulphonylurea known to block K_ATP_ channels [14], [15] and to promote gap junctions formation and coupling between *β*-cells [16], [17], [18], increased the proportion of synchronized cells, and decreased that of non synchronous and silent cells (Fig. 2 *A*). Conversely, quinine, an antimalarial known to block a subpopulation of K^+^ channels [19], [20] and to decrease Cx36-dependent coupling [21], decreased the proportion of synchronous cells (Fig. 2 *B*). These data show that MIN6 cells are a convenient model to test for the presence and extent of Cx36 coupling, and that this coupling may be evaluated by the proportion of cells featuring synchronized Ca^2+^ transients.

**Table 1:** Coupling of MIN6 cells, as investigated by tracer microinjection.

MIN6 type	treatment	coupling extent#		coupling incidence (% injections)	
		LY	EB	LY	EB
**Cx36 antisense**	none	1.28±0.11***n = 18	1.48±0.13****n = 25	27.8	40.0
**Wild type**	none	2.58±0.39 n = 19	3.20±0.32 n = 24	73.3	80.0
**Wild type**	DMSO	2.50±0.26 n = 24	3.17±0.29 n = 23	73.7	82.6
**Wild type**	glibenclamide	4.64±0.53****n = 11	5.83±0.49****n = 12	81.8	91.3
**Wild type**	norcantharidin	3.96±0. 42***n = 23	4.15±0.50*n = 20	82.6	85.0
**Wild type**	zaprinast	4.08±0.45***n = 22	4.15±0.44*n = 26	81.8	88.5
**Wild type**	quinine	1.70±0.26****n = 10	1.82±0.26****n = 11	50.0	54.5
**Wild type**	mebeverine	1.79±0.21****n = 14	2.29±0.25*n = 21	57.1	66.7
**Wild type**	gedunin	1.96±0.18****n = 24	2.26±0.27**n = 19	66.7	68.4

Coupling extent  =  number of cells labeled by the microinjected tracer; coupling incidence  =  percent of injections showing coupling;

# Data are mean ± SE; n  =  number of microinjections. LY  =  Lucifer yellow; EB  =  ethidium bromide.

* p<0.05, **p<0.01, ***p<0.007, ****p<0.001 as compared to untreated wild type controls by the median test.

### The Evaluation of the Ca^2+^ Synchronization can be Automated

To simultaneously monitor changes in [Ca^2+^]_i_ in large numbers of MIN6 cells, we used an imaging system which allows for automatic fluorescence recordings. These recordings were analyzed by a dedicated program which allows to calculate a “synchrony index” (see Figs. S2 and S3 and Materials S1). To validate this program, we first revisited WT and AS MIN6 cells, using in parallel the confocal approach mentioned above (Fig. 2 *C*) and the semi-automatic approach outlined in Fig. 2 *D*. Both approaches showed that the synchrony index of WT cells was higher than that of AS MIN6 cells, and was increased after exposure of WT cells to glibenclamide (Figs. 2 *C* and *D*). However, the automated approach provided for the analysis of much larger numbers of cells in a much shorter time period. The data document that the measurement of the intercellular synchronization of Ca^2+^ transients can be adapted for medium-scale screenings, and is affected by drugs altering cell-to-cell coupling.

### Various Molecules Affect the Intercellular Synchronization of Glucose-induced Ca^2+^ Transients

**Figure 3:**
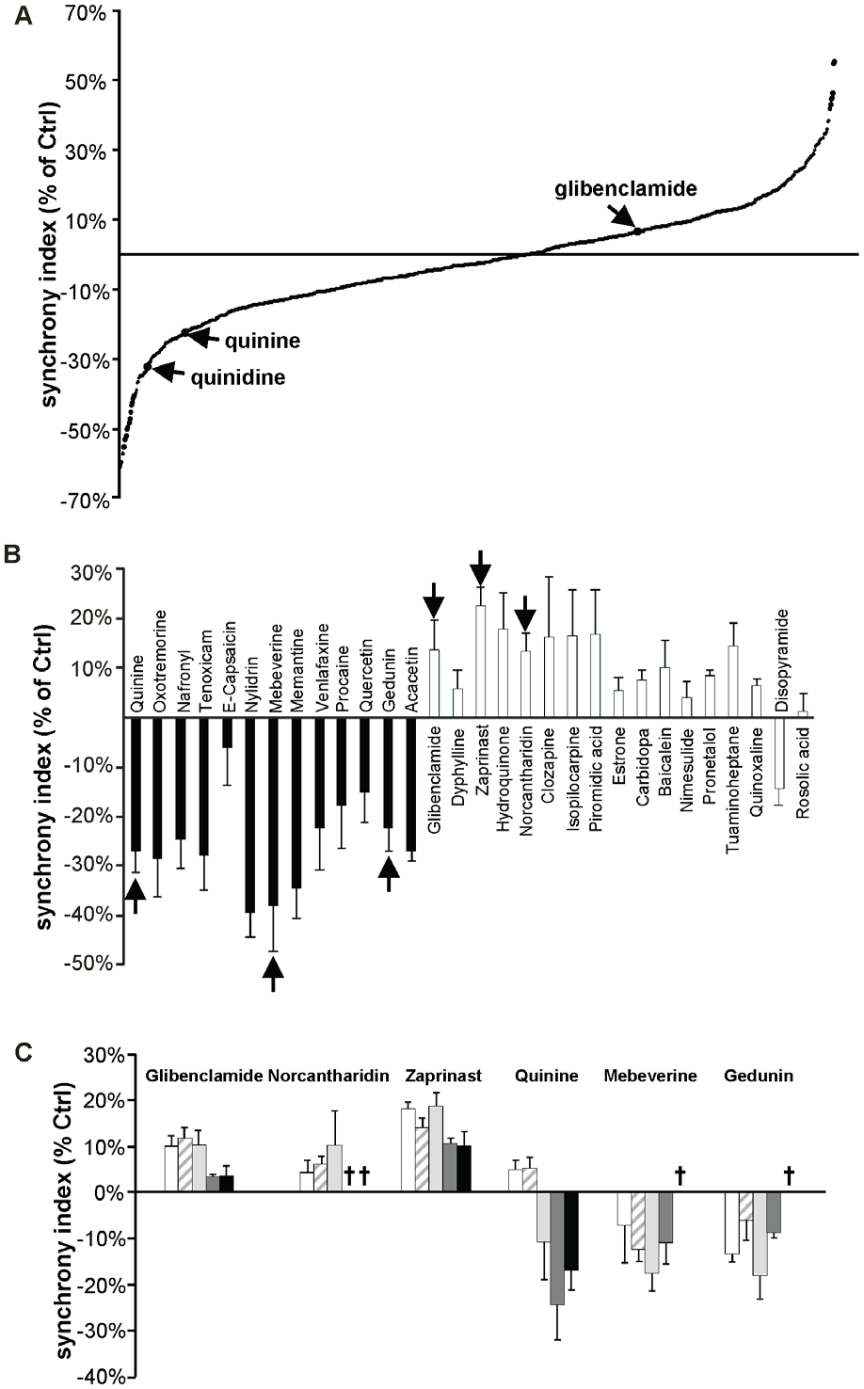
Primary and secondary screenings identify several molecules affecting the synchrony index of MIN6 cells. (*A*) The primary screening of 1040 compounds revealed that several molecules increased or decreased the synchrony index. Each point of the curve shows the percentage change in synchrony index induced by one drug, relative to the value evaluated in WT MIN6 cells exposed to DMSO, which was the vehicle of all compounds, and served as internal control. Of note, glibenclamide increased the synchrony index by ∼12%, whereas quinine, and its stereoisomer quinidine, decreased it by 22 and 34%, respectively; (*B*) 17 drugs increasing (*white bars*) and 13 drugs decreasing the synchrony index (*black bars*) were repeatedly tested, and their effect on the synchrony index plotted relative to that of WT MIN6 cells exposed to DMSO. 97% of the drugs reproduced the effects observed in the primary screening. The six drugs pointed by the arrows were selected for further experiments. Data are means ± SE of four independent experiments; (*C*) Dose-response experiments were conducted with the six selected drugs. The effect of these drugs on the synchrony index was plotted relative to that of WT MIN6 cells exposed to DMSO. The effects on the synchrony index varied with the concentration tested. Glibenclamide, norcantharidin and zaprinast increased the synchrony index for concentrations ≤10 µM, whereas quinine, mebeverine and gedunin decreased it. Several drugs showed cytotoxicity at concentrations >10 µM. All drugs were tested at the following concentrations: 0.1 µM, 1 µM, 10 µM, 50 µM and 100 µM. “**†**” indicates concentrations toxic for MIN6 cells, as evaluated by cell detachment from the support. Data are means ± SE of at least four independent experiments.

Exposure of MIN6 cultures to a collection of 1040 drugs, tested at 10 µM for 24 h, identified 449 molecules (43.2%) that had no effect (defined as a change in synchrony index < than 10% of control value), 183 molecules (17.6%) that increased this parameter, and 270 molecules (26%) that decreased it (Fig. 3 *A*). Among the 1040 molecules, 138 (13.2%) were found to be toxic on MIN6 cells as evaluated by cell detachment from the support. In agreement with previous data, glibenclamide was among the drugs increasing the synchrony index, whereas quinine and its analog quinidine were among the drugs that decreased it (Fig. 3 *A*).

Based on this primary screening, we selected the drugs which increased the synchrony index by more than 30% (17 compounds) and those which decreased this index by more than 40% (13 compounds). In a series of four independent experiments, 29 of these drugs (97%) showed reproducible effects, similar to those observed during the primary screening (Fig. 3 *B*). These experiments indicate that several drugs consistently modulate the intercellular synchronization of glucose-induced Ca^2+^ transients, and that the method is reproducible. Several experiments were repeated over a three year period of time, i.e. with MIN6 cells differing in passage number (71–122 passages), without showing major qualitative or quantitative differences in synchrony index, the main parameter which we evaluated in this study. Dose-response experiments with the 30 drugs that had been selected for the secondary screening (Fig. 3 *C* and Fig. S4), showed a variable dose-dependence of the synchrony index changes. In most cases, the 10 µM concentration that had been chosen for most experiments induced the largest changes in synchrony index, without affecting cell viability (Figs. 3 *C* and S4). The data indicate that the screening procedure was sensitive enough to detect dose-dependent effects for most drugs, and further validate the use of the 10 µM concentration for most of the experiments.

### Drugs Altering the Synchrony Index Modulate Cx36 and Coupling of MIN6 Cells

**Figure 4:**
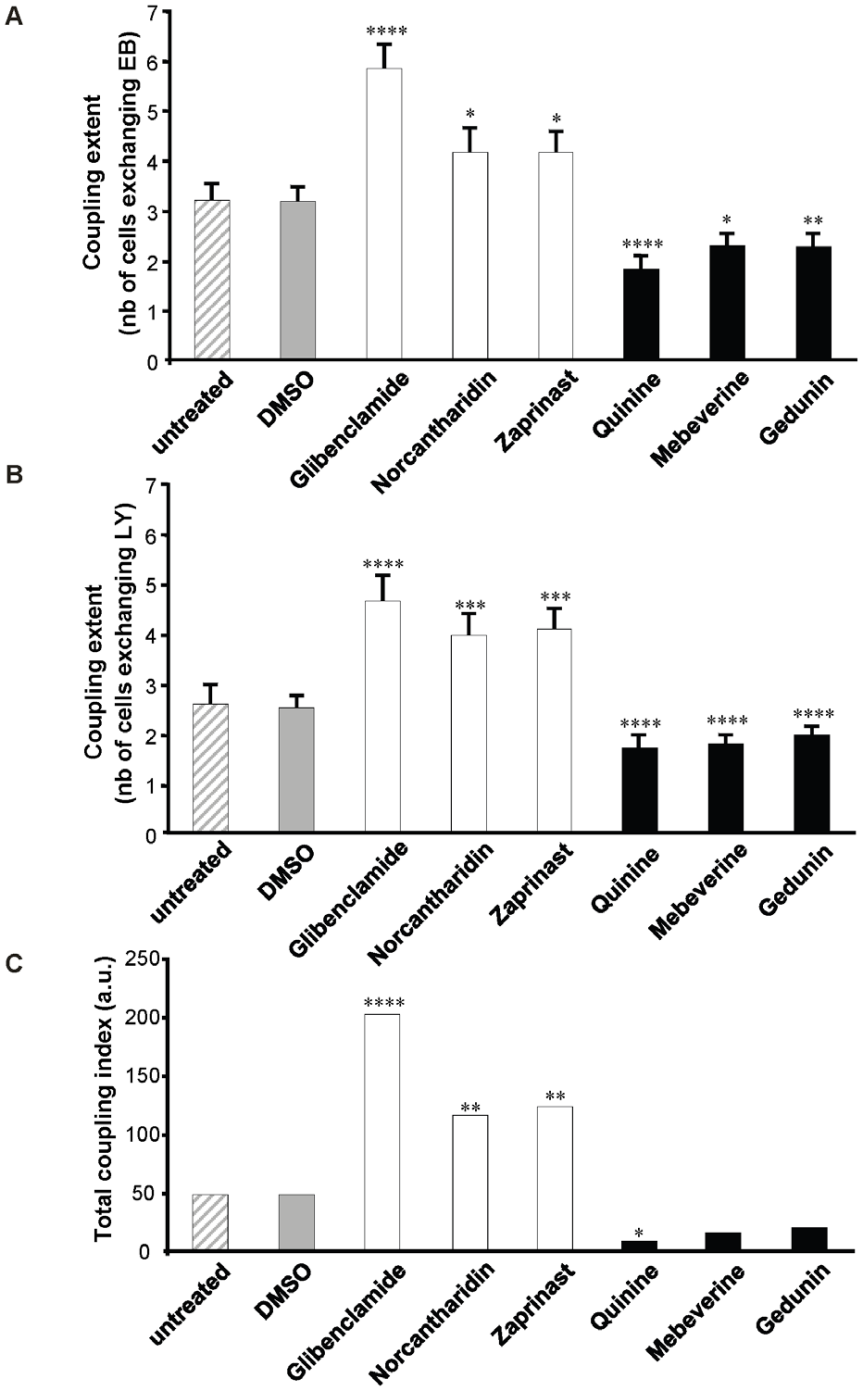
Molecules altering the synchrony index, modify Cx36-dependent coupling. (*A*–*B*) Microinjection of either EB (*A*) or LY (*B*) revealed a similar extent of coupling in MIN6 cells exposed to DMSO (*gray bars*), the solvent used to dissolve all drugs, and untreated controls (*hatched bars*). All the drugs increasing the synchrony index (*white bars*) increased the extent of coupling, whether the permeability of Cx36 channels was tested with EB (*A*) of LY (*B*). In contrast, all the drugs decreasing the synchrony index (*black bars*) also decreased the extent of coupling tested with either EB (*A*) or LY (*B*). Data are means + SE of the number of injections indicated in Table 1. *p<0.05, **p<0.01, ***p<0.001, ****p<0.0007 versus values obtained in cells exposed to DMSO; (*C*) Computing of total coupling index, assuming independent permeability of Cx36 channels to EB and LY, showed that coupling was significantly increased by all the drugs enhancing the synchrony index (*white bars*) and reduced by all the drugs decreasing this index (*black bars*). *p<0.05, **p<0.01, ****p<0.0007 versus values obtained in cells exposed to DMSO, using a chi-square test on the number of injections indicated in Table 1.

From the secondary screening, we selected two drugs that most efficiently increased (zaprinast) or decreased (mebeverine) the synchrony index, as well as two drugs (norcantharidin and gedunin) which affected this parameter to a similar extent than glibenclamide and quinine, respectively (Fig. 3 *B*). At the 10 µM concentration, the six drugs showed effects that were reproducible, and consistent with those of the secondary screening (Fig. 3 *C*), without inducing cell death (Fig. S5). To test whether the effects of these drugs on the synchrony index correlated with their effects on cell-to-cell coupling, clusters of MIN6 cells were microinjected with either LY or EB [5], [16] 24 h after exposure to 10 µM of each of these six drugs. MIN6 cells exposed to 0.1% DMSO showed an extent (Fig. 4 and Table 1) and an incidence of coupling (Fig. S6 and Table 1) comparable to that of untreated controls. In comparison, cells exposed to either glibenclamide, zaprinast or norcantharidin showed a significant increase in the extent of coupling, whether this was tested with LY or EB (Fig. 4 and Table 1). In contrast, cells exposed either to quinine, gedunin or mebeverine showed a significant decrease in the extent of coupling with either tracer (Fig. 4 and Table 1).

**Figure 5:**
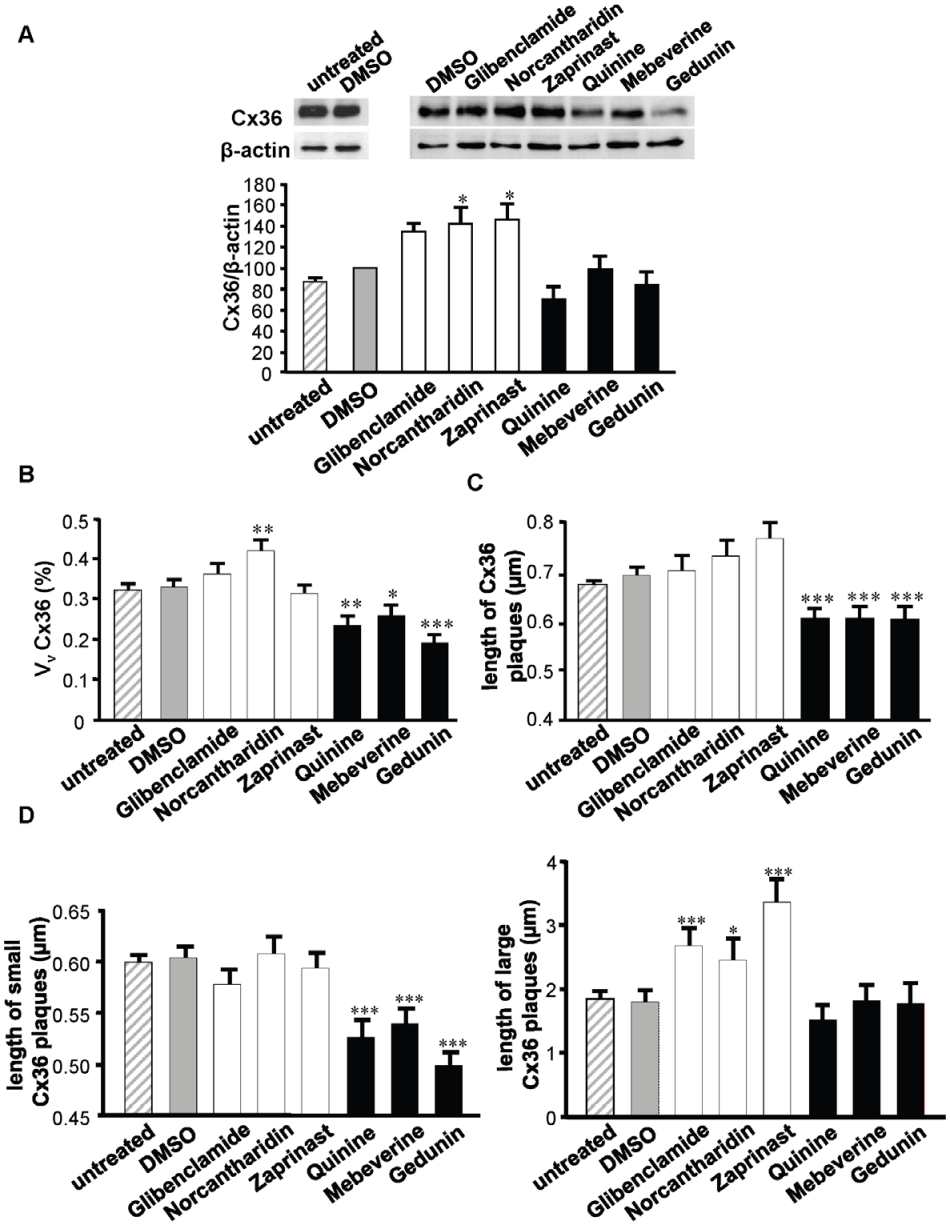
Molecules altering the synchrony index, modify Cx36 expression and the size of Cx36 plaques. (*A*) Immunolabeling of total protein extracts revealed that the steady state levels of Cx36 were increased in the MIN6 cells exposed to compounds enhancing the synchrony index, but unchanged in the MIN6 cells exposed to compounds reducing this index. 20 µg proteins were loaded per lane. The bars show means + SE ratio of the Cx36 to the *β*-actin signal, as evaluated by densitometric analysis of 5 independent experiments. *p<0.05 versus DMSO-exposed controls; (*B*) The volume density (Vv) of Cx36 was increased by some of the drugs that enhanced MIN6 cell synchronization, and decreased by all the drugs decreasing this synchronization. Data show means + SE of three independent experiments. *p<0.05; **p<0.01; ***p<0.001 versus DMSO-exposed controls; (*C*) The Vv changes were mostly accounted for by differential alterations in the length of Cx36 plaques, defined as the span of the longest chord through the object outline. Data show means + SE of three independent experiments. *p<0.05; **p<0.01; ***p<0.001 versus DMSO-exposed controls; (*D*) The length of “small” Cx36 plaques was reduced in cells exposed to drugs decreasing the synchrony index (*left panel*), whereas that of “large” plaques increased in cells exposed to drugs enhancing this index (*right panel*). Data show means + SE of three independent experiments. *p<0.05 and ***p<0.001 versus DMSO-exposed controls. For all parameters, no difference was observed between untreated and DMSO-treated cells.

To test whether the changes in coupling correlated with changes in Cx36 expression, we immunoblotted total cell extracts. We found that, norcantharidin and zaprinast significantly increased the levels of Cx36, whereas mebeverine and gedunin did not (Fig. 5 *A*). By immunolabeling, norcantharidin was found to significantly increase the volume density of the immunostained Cx36, whereas all the drugs decreasing the synchrony index reduced this volume density (Fig. 5 *B*). These changes were mostly accounted for by a change in the size of Cx36 plaques, which was decreased after exposure to all the drugs reducing the synchrony index (Fig. 5 *C* and *D*). These results indicate that the drugs we choose for their effects on the intercellular synchronization of Ca^2+^ transients affected the function and distribution of Cx36 in MIN6 cells.

### Drugs Altering Coupling and Synchrony Index Modulate the Secretory Function of MIN6 Cells

**Figure 6:**
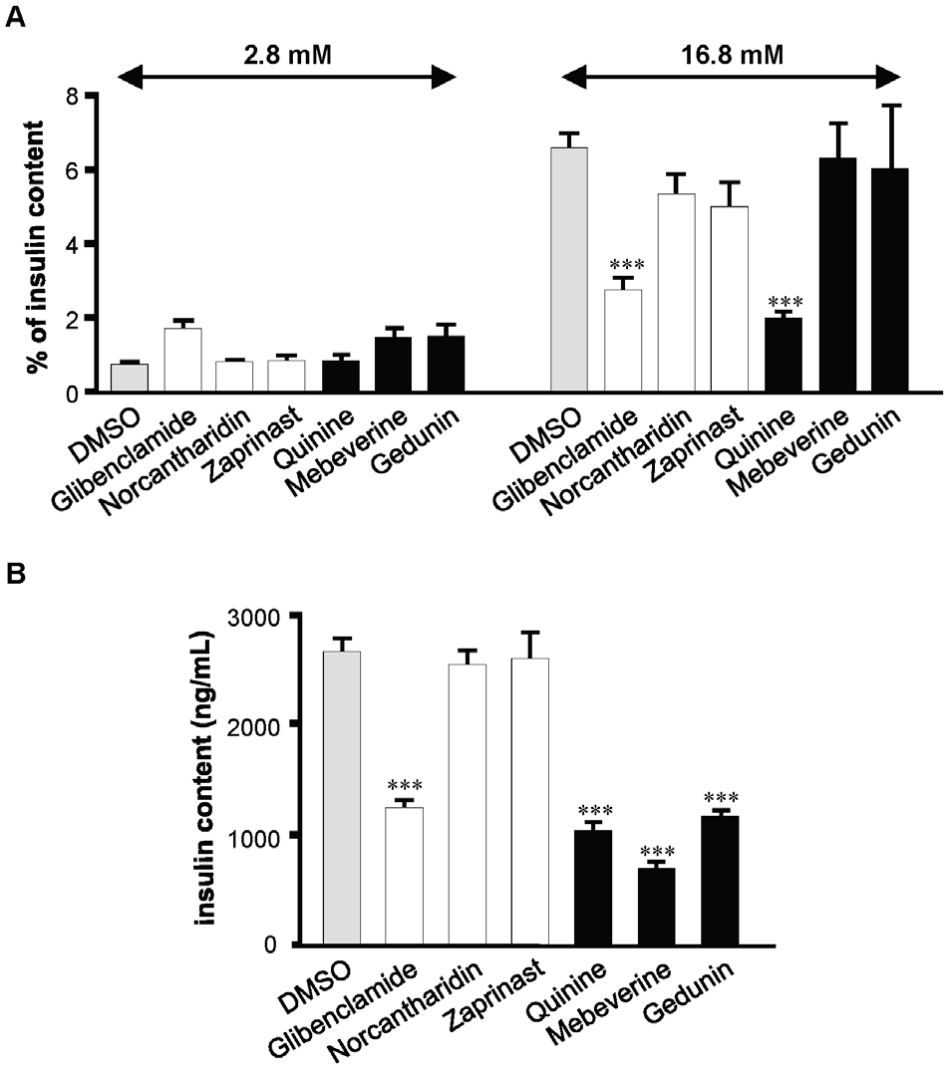
Drugs altering the synchrony index modulate the function of MIN6 cells. (*A*) Insulin release was tested during a 30 min exposure to either 2.8 mM (*left panel*) or 16.8 mM glucose (*right panel*) in cells that had been exposed for 24 h to 10 µM the drugs selected in the secondary screening. No drug significantly altered the basal insulin release (*left panel*). All drugs, but glibenclamide and quinine, also increased to control levels their fractional insulin release (*expressed as percentage of insulin content*), when challenged with a high glucose level (*right panel*). (*B*) The insulin content of MIN6 cells was reduced by all drugs decreasing the synchrony index and by glibenclamide. Data are means + SE of three independent experiments. ***P<0.001 versus DMSO-exposed controls.

To assess whether the drugs altering the coupling and synchrony index modulated the function of MIN6 cells, we investigated their effects on the release and content of insulin under the conditions (24 h exposure to 10 µM) which were used for the other experiments. In the presence of a non stimulatory concentration of glucose (2.8 mM), the basal release of insulin was normally low (∼1–2% of the content), and alike in all groups (Fig. 6 *A*). Increasing the concentration of the sugar to 16.8 mM, raised by ∼7 fold the secretion of both controls and cells exposed to norcantharidin, zaprinast, mebeverine and gedunin (Fig. 6 *A*). The data show that these drugs did not block glucose-stimulated insulin release, confirming that they had no deleterious effects on MIN6 cell function.

We further evaluated insulin content, another important aspect of insulin secretion, and found that it was significantly reduced by the three drugs that decreased the synchrony index, whereas it remained at control levels in the cells exposed to norcantharidin and zaprinast, two drugs increasing this index (Fig. 6 *B*). The data indicate that the newly identified drugs that increased (norcantharidin and zaprinast) and decreased the synchrony index (mebeverine and gedunin) had an opposite effect on the insulin content of MIN6 cells. The alteration of this secretory parameter was specific, inasmuch as the same drugs did not modify the insulin release of the very same cell cultures. This observation is not contradicted by the finding that MIN6 cells exposed for 24 h to either glibenclamide or quinine showed reduced insulin release and content (Fig. 6 *A* and *B*), in spite of opposite effects on the synchrony index. Rather, these findings are consistent with previous reports documenting that the two drugs have a long lasting stimulatory effect on insulin release while blocking insulin production [20], [22], [23]. The data stress the complexity of the regulatory network in which multiple mechanisms, including Cx36 signaling, concur to finely tune insulin secretion.

## Discussion

Cx36 signaling contributes to *β*-cell function [11], [12], [24], [25], and drugs modulating this signaling are required to unravel the mechanisms underlying the Cx36-dependent alterations, as well as to develop novel targeted pharmacological tools to correct these alterations. Glibenclamide and quinine derivatives have already been reported to modulate Cx36 gap junctions of *β*-cells [17], [18], [21], but in no case the effects of these drugs are direct and specific. To screen for novel, more specific drugs, we took advantage of the synchronization of glucose-induced Ca^2+^ transients between pancreatic *β*-cells, which is largely dependent on Cx36 [3], [4], [6]. Using an innovative approach that provides for a non invasive, semi-automatic and fast, still indirect evaluation of Cx36-dependent coupling, we found a number of molecules which significantly, and consistently modified the intercellular synchronization of Ca^2+^ transients, usually in a dose-dependent way.

We chose four drugs, which consistently increased (zaprinast, norcantharidin) or decreased the synchrony index (mebeverine, gedunin), at either maximal (zaprinast, mebeverine) or intermediate levels (norcantharidin, gedunin), and that have not yet been documented for effects on either Cx36 or insulin-producing cells. We document that these drugs altered MIN6 cell coupling, as anticipated by the changes in synchrony index, presumably as a result of a redistribution of Cx36 within the cell membrane. We further show that the drugs decreasing the synchrony index and uncoupling MIN6 cells, also reduced the insulin content of MIN6 cells, consistent with the previously reported relationship between the levels of expression of *Gjd2* (the gene coding for Cx36) and the insulin genes [25], as a result of a common regulation of the cognate promoters by at least the transcription factor beta2/Neurod1 [26]. Previous studies have also shown that loss of Cx36 prevents glucose-stimulated insulin release, but that this effect is not observed till more than 50% of the native protein is lost [4], [6]. Again, the results of our study are fully consistent with these previous findings since they show that drugs which partially uncoupled MIN6 cells did not alter the insulin release induced by a high glucose concentration. With evolution, the secretory function of *β*-cells has become under the control of multiple mechanisms, whose partial overlapping and interactions strive to maintain the vital biosynthesis and release of insulin, even when individual control pathways are compromised [25]. Cx36-dependent signaling is part of this complex system, whose hierarchical organization [4], [5], [6], [25] remains largely obscure. The new pharmacological tools, identified here, may help deciphering this hierarchy. It is most unlikely that a simple, not to mention linear relationship is found between the synchrony index, and the extent of either coupling or insulin secretion. The complexity of the relationship between these parameters is exemplified by our findings with glibenclamide and quinine derivatives, whose effects vary with exposure time, consistent with previous reports [20], [22], [23], and which is due to the pleiotropic effects these drugs have on multiple key effectors of *β*-cell coupling and insulin secretion. Further work is required to test whether the new drugs we have identified here also have other effects beside that we document here on Cx36-dependent signalling.

In summary, we report on a novel procedure for the non invasive investigation of Cx36-dependent coupling, which allows for the identification of hitherto unknown molecules affecting Cx36. This identification is instrumental for elucidating the mechanisms whereby Cx36 affects the function of the several cell types, including *β*-cells, neurons and neuron-derived endocrine cells, which are coupled by this protein [25], [27]. In turn, this may lead to the development of innovative and targeted therapies for the numerous patients with diseases that are linked to altered function of the Cx36-coupled cells.

## Materials and Methods

### Cell Lines

MIN6 cells [28] at passages 71–122, were cultured in Dulbecco’s modified Eagle’s medium (GIBCO-Invitrogen) containing 25 mM glucose, and supplemented with 15% heat inactivated FCS, 70 µM *β*-mercaptoethanol, 110 U/mL penicillin and 110 µg/mL streptomycin [4], [5]. Cells were plated at the initial density of 5×10^5^ cells/mL, and grown for 3 days at 37°C in a humidified incubator, which was gassed with air and CO_2_ (7%) to maintain pH at 7. At the time of the experiments, cultures had a density of about 10^6^ cells/mL. The passages used for specific experiments are indicated below.

### Transfection of a Cx36 Antisense Construct

The full coding sequence of mouse Cx36 was inserted in an antisense orientation, using two EcoRI sites, within plasmid pcDNA3 (Invitrogen), which contains the CMV early promoter region and a neomycin-resistance gene. Subconfluent cultures of MIN6 cells (passage 71) were transfected with Polyfect Transfection reagent (Qiagen), as per the manufacturer’s instructions, and selected in the presence of 350 µg/mL Geneticin (G-418 sulfate; GIBCO BRL-Invitrogen). After 2 weeks of selection, single cells were plated in 96-well plates, one cell per well, to allow for cloning of stable transfectants.

### Cx36 Expression and Distribution

Total protein extracts of wild type and antisense-transfected cells were prepared as reported [29]. Protein concentration was measured using a colorimetric assay (BCA Protein Assay Reagent Kit, Thermo Scientific), to load 20 µg aliquots per lane. Electrophoresis, transfer and signal development were performed as previously described [29]. Briefly, membranes were incubated overnight at room temperature with an affinity-purified rabbit serum against Cx36 [2], diluted 1∶200, or for 2 h with a mouse monoclonal antibody against smooth muscle *β*-actin (MAB1501; Chemicon International), diluted 1∶10,000, and then exposed for 60 min at room temperature to either a goat anti-rabbit Igs, conjugated to horseradish peroxidase (Bio-Rad Laboratories) and diluted 1∶5,000 for Cx36 immunolabeling, or to a goat anti-mouse Igs, conjugated to horseradish peroxidase (Bio-Rad Laboratories), and diluted 1∶3,000 for *β*-actin immunolabeling. Cx36 levels were evaluated by densitometric analysis of the films using the Quantity One 4.5-2 software (Bio-Rad Laboratories) and expressed relative to those of *β*-actin, which served as internal standard.

To measure Cx36 distribution, MIN6 cell monolayers grown for 3 days on glass coverslips were either permeabilized for 3 min in −20°C acetone (Cx36) or fixed for 10 min in 4% paraformaldehyde (GLUT-2). Immunostaining was then performed as previously described [2], [4], [6], including on cryosections of mouse pancreas that served as controls for proper Cx36 and GLUT-2 immunostaining. MIN6 cells were exposed for 2 h at room temperature to a rabbit polyclonal antibody against either Cx36 (Zymed-Invitrogen) diluted 1∶60, or amino acids 512–523 of GLUT-2, diluted 1∶50 [30]. After exposure to fluorescein-conjugated antibodies against rabbit Igs, diluted 1∶500 (Boehringer Mannheim Biochemica), cells were counterstained with 0.03% Evans blue, coverslipped and pictures were taken with an Axiophot fluorescence microscope (Zeiss). To evaluate Cx36 distribution, images were analysed using the Metamorph/MetaXpress software (Molecular Devices). Briefly, the outline of each MIN6 cell cluster was drawn by hand on projections of the digitized images, and its area scored by the program. In a second step, fluorescent objects overlying each cluster were automatically selected and counted. Values of number, length and areas of objects were expressed relative to cluster area, to provide average estimates of numerical (Nv) and volume density (Vv). MIN6 cells at passages 97–103 were used for these immunolabellings.

### Drugs

A collection of 1040 FDA-approved and biologically active compounds (NINDS Custom Collection™, MicroSource Discovery Systems) was tested. Each compound was dissolved in DMSO and used at a concentration of 10 µM for 24 h at 37°C. This concentration was chosen based on a previous study using the same drug library [31], and on standard protocols for primary screening of small organic molecules [32], [33]. For the establishment of dose-response curves, drugs selected after the primary screening were tested for 24 h at concentrations ranging from 0.1 to 100 µM. The synchrony index was evaluated for each concentration.

### Cell Viability

Cells exposed for 24 h to the drugs under test, were washed with culture medium devoid of phenol red (GIBCO-Invitrogen), and loaded, in this medium, with 4 µM calcein/AM, 8 µM ethidium bromide (Live/Dead® Viability/Cytotoxicity; Molecular Probes) and 1.6 µM Hoechst (Molecular Probes). Images of four regions of interest (ROIs) were taken per well using the ImageXpress Micro plate reader. Percentage of dead cells (red fluorescence) was calculated relative to total cell number (blue fluorescence), using the nucleus counting functionality of the ImageXpress software (Molecular Devices). MIN6 cells used for toxicity experiments were between passages 84 and 89.

### Ca^2+^ Measurements

In a first series of experiments, MIN6 cultures were plated at a density of 3×10^5^ in a 35 mm dish and grown for 3 days on glass coverslips, and then exposed to a KRB buffer (125 mM NaCl, 5.7 mM KCl, 1.2 mM MgCl_2_, 1.2 mM CaCl_2_ and 10 mM Hepes, pH 7.4), supplemented with 5 µM Fluo-3/AM and 0.02% (w/v) the non-ionic detergent pluronic acid (Molecular Probes), 0.1% BSA (fraction V; Sigma Chemical) and 2 mM glucose. After 1 h incubation at 37°C, cells were transferred for 10–15 min in KRB containing 20 mM glucose and 15 mM tetraethylammonium (TEA) [4]. Using a NIPKOW scanning confocal system (QLC 100, Visitech International), mounted on an inverted Axiovert 200 M microscope (Zeiss), cells were monitored using a 40×, NA = 1 oil immersion objective (Zeiss), an excitation wavelength of 488 nm, and an emission wavelength of 526 nm. Changes in fluorescence intensity reflecting oscillations in [Ca^2+^]_i_ were simultaneously measured in 5–15 cells per cluster at 37°C, and acquired using a 520 nm long-pass filter and a Cascade II 16 bits cooled EMCCD frame transfer camera (Photometrics-RopperScientific). To estimate the synchrony of Ca^2+^ transients among the cells of each cluster, relative fluorescence levels were plotted as a function of time. Synchrony was defined as the percentage of cells of each cluster that displayed at least 70% simultaneous [Ca^2+^]_i_ transients, during a minimum of 10 oscillations. Other oscillating cells were considered to be “asynchronous”. Cells which did not respond to the glucose stimulation were considered to be “silent”. MIN6 cells used for this first series of experiments were between passage 71 and 74.

In a second series of experiments (Fig. S7), MIN6 cells were plated at a density of 2×10^4^ cells per well of a 96-well plate, and grown for two days before drug treatment (10 µM for 24 h). Three day-old cultures were loaded with Fluo-3 as described above and transferred onto a reader fitted on an inverted and fully automated epifluorescence microscope, equipped with a 10× SFluor, NA = 0.45 objective (Nikon), and a Photometrics CoolSNAP HQ 12-bits digital interline CCD camera (ImageXpress Micro, Molecular Devices). Images were sequentially recorded (75 time points at a 1 sec interval) in ROIs which comprised multiple MIN6 clusters (40–50 clusters or 200–1000 cells per ROI), within each plate well (Movie S1). Fluorescence intensity was plotted as a function of time using the ImageXpress software. To estimate the synchrony of Ca^2+^ transients in each well, an average “synchrony index” was generated by a dedicated, semi-automatic program described in the Supporting Material.

In the primary screening, 1040 compounds were tested once under the conditions described above. In the secondary screening, 17 drugs which most potently increased the synchrony index and 13 drugs that decreased this parameter were repeatedly tested. In parallel, a dose-response curve was established for these drugs, by testing each of them at 0.1–100 µM concentrations. In the secondary screening, the dose-response curves of the 30 selected drugs, were tested in four independent experiments. MIN6 cells at passages 76–86 and 83–89 were used for the primary and secondary screening, respectively. Dose-response experiments were run with MIN6 cells at passages 120–122.

### Cell Coupling

Three day-old cultures of MIN6 cells were transferred onto the heated (37°C) stage of an inverted Zeiss ICM35 microscope, and individual cells were microinjected with either 4% Lucifer Yellow or 4% ethidium bromide in 10 mM Hepes-buffered (pH 7.2) 150 mM LiCl, as previously reported [13], [16], [34]. Cell coupling extent was calculated after each injection by scoring the number of cells containing one of the two injected tracers. Cell coupling incidence was determined by calculating the percentage of injections resulting in the cell-to-cell transfer of one of the two tracers (i.e. more than one cell stained). MIN6 cells used for coupling experiments were between passages 94 and 96.

### Insulin Secretion

Cells were plated at a density of 7×10^5^ cells in 2 mL medium within 35-mm Petri dishes, cultured for 2 days, and then exposed to 10 µM of the different drugs for 24 h. After washing, cells were preincubated 30 min at 37°C in a KRB buffer (133.4 mM NaCl, 5 mM NaHCO_3_, 4.7 mM KCl, 1.2 mM KH_2_PO_4_, 2.4 mM MgSO_4_, 3.4 mM CaCl_2_, 10 mM Hepes and 0.5% BSA pH 7.4) containing 2.8 mM glucose, and eventually incubated for 30 min in the same medium supplemented with different concentrations of glucose (2.8 or 16.8 mM), and the drugs under test. The medium was then collected, centrifuged for 20 min at 4°C and at 3,300 *g*, and the supernatant frozen at −20°C for insulin release measurements. The cultures were extracted overnight at 4°C in acid-ethanol, and the extracts were also frozen for determination of total insulin content. Insulin was measured by radioimmunoassay with a charcoal separation step, using rat insulin as standard and guinea pig anti-rat insulin serum as antibody (Linco Research, Inc.). Values of secreted insulin were expressed as percentage of the cell content. MIN6 cells used for insulin secretion experiments were between passages 92 and 94.

### Data Analysis

Data are expressed as means + SE of the indicated number of experiments. Statistical analysis was performed using the Statistical Package for Social Science (SPSS 15.0, SPSS inc.). For normally distributed values, differences between means were assessed by analysis of variance, using the *post hoc* Bonferroni test. For asymmetrically distributed values, differences between distributions were assessed by the Mann-Whitney and the Kolmogorov-Smirnov tests. Coupling extent data were compared using the median test. Differences were considered significant when p<0.05.

## Supporting Information

Figure S1 **The intercellular synchronization of Ca^2+^ oscillations correlates with Cx36 expression of MIN6 cells.** (*A, upper panel*) During stimulation by 20 mM glucose and 15 mM TEA, most WT MIN6 cells, which express native levels of Cx36, show synchronous Ca^2+^ oscillations (*traces of different colours are recorded in different cells*). (*A, lower panel*) In contrast, most AS MIN6 cells, which express reduced levels of Cx36, show asynchronous Ca^2+^ transients; (*B*) Quantification revealed that the proportion of synchronous cells was higher in WT (*black bars*) than AS MIN6 cells (*open bars*), whereas the reverse was true for both asynchronous and silent cells. Data are means + SE of three independent experiments. *p<0.05, **p<0.01 and ***p<0.001 for AS versus WT MIN6 cells.
(TIF)

Figure S2 **Processing of Fluo-3-loaded MIN6 cells, for evaluation of intercellular Ca^2+^ synchrony.** (*A*) Low magnification view of clusters of Fluo-3-loaded MIN6 cells, as seen under green fluorescence illumination in the ImageXpress equipment; (*B*) The software automatically detects clusters comprising more than five cells (*green*), outlines (*yellow line*), and identifies them by a number. Clusters of less than five cells are identified separately (*white*); (*C*) Clusters are sorted by size, and those containing less than five cells discarded from subsequent calculations; (*D*) Higher magnification view of one cluster of nine MIN6 cells, featuring a green fluorescence due to Fluo-3 uptake; (*E*) The same cluster is seen under a rhodamine channel, which detects the regions of highest fluorescence intensity. Deconvolution improves cell detection; (*F*) A region of five pixel width (*yellow line*) is automatically defined around each nuclear region to define the ROIs where fluorescence intensity was recorded as a function of time. Bar, 50 µm in A, B and C, and 10 µm in D, E and F.
(TIF)

Figure S3 **Steps for the automatic evaluation of the “synchrony index”.** (*A*) Records of fluorescence intensity as a function of time are shown for a fully synchronized (*left column*) and a poorly synchronized cluster (*right column*). Each colour shows the recording from a different cell (for clarity reason, only three cells were selected for these illustrations); (*B*) The envelope of each curve was drawn using a morphological opening and closing operation, resulting in a lower local minimum (*black dotted line*) and an upper local maximum bonds, respectively (*coloured dotted lines*); (*C*) The amplitude of each curve was set to 1, to give each cell recording the same weight; (*D*) An average equalized curve was computed for each cluster; (*E*) The presence of peaks was detected by computing the derivative of each curve; (*F*) The average peak values of all the clusters estimated the “synchrony index”, an indicator of the average synchrony of all the MIN6 cells screened in a well.
(TIF)

Figure S4 **Dose-response relationship of the 24 drugs selected from the primary screening.** The effect on synchrony index (relative to that of WT MIN6 cells exposed to DMSO) was plotted as a function of 0.1, 1, 10, 50 and 100 µM concentration of each drug. Data are means ± SE of at least four independent experiments. **†**indicate toxic concentrations, as evaluated by cell detachment from the support.
(TIF)

Figure S5 **Drugs affecting the synchrony index and Cx36 coupling, and selected from the secondary screening, were not toxic to MIN6 cells.** The percentage of dead cells, after a 24 h treatment with 10 µM the different drugs selected from the secondary screening, was comparable to that observed in DMSO-exposed controls. No difference was observed between untreated and DMSO-treated cells. Data are means + SE of three independent experiments.
(TIF)

Figure S6 **Coupling incidence of MIN6 cells, as investigated by tracer microinjection.** (*A*) The coupling incidence (i.e. the percentage of injections showing cell-to-cell transfer of a tracer) of both Ethidium Bromide (*EB, left panel*) and Lucifer Yellow (*LY, right panel*) was markedly reduced in AS MIN6 cells when compared to WT cells. Values are means of four experiments. (*B*) The coupling incidence of both EB (*upper panel*) and LY (*lower panel*) was not markedly affected by the six drugs tested, even though a trend towards an increase and a decrease was observed in the presence of drugs increasing (*white bars*) and decreasing the synchrony index (*black bars*), respectively.
(TIF)

Figure S7 **Schematic of the screening protocol.** Flowchart of the Ca^2+^ imaging screening protocol. Day 0: Plating of MIN6 cells in 96-well plate format; Day 2: Compound treatment (10 µM, 24 h); Day 3: Ca^2+^ imaging; Day 4: Ca^2+^ imaging analysis.
(TIF)

Movie S1 **Glucose-induced Ca^2+^ oscillations are synchronous in cultures of WT MIN6 cells.** Video recording of one cluster of Fluo-3 loaded WT MIN6 cells during stimulation by 20 mM glucose and 15 mM TEA, revealed Ca^2+^ oscillations. Within each cluster, these oscillations were synchronous between most cells.
(AVI)

Materials S1 **Algorithms for the evaluation of intercellular Ca^2+^ synchronization in large MIN6 cell populations.**
(DOCX)
