# Supplementary figures and images for: The Intercellular Synchronization of Ca2+ Oscillations Evaluates Cx36-Dependent Coupling
Source: PLoS One. 2012 Jul 25;7(7):e41535. doi: 10.1371/journal.pone.0041535 (PMC3405138; doi:10.1371/journal.pone.0041535)

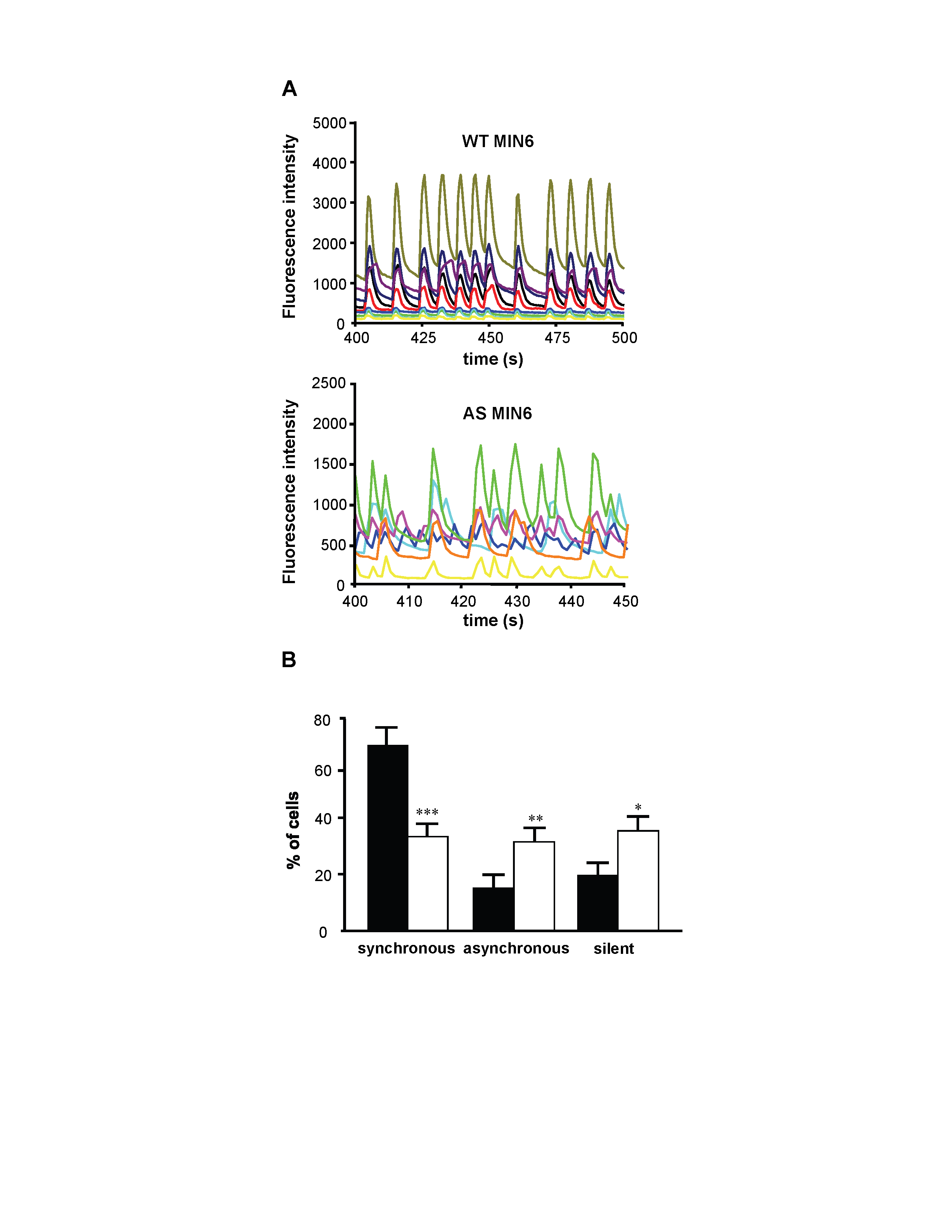

Supplement: Figure S1 — The intercellular synchronization of Ca2+ oscillations correlates with Cx36 expression of MIN6 cells. (A, upper panel) During stimulation by 20 mM glucose and 15 mM TEA, most WT MIN6 cells, which express native levels of Cx36, show synchronous Ca2+ oscillations (traces of different colours are recorded in different cells). (A, lower panel) In contrast, most AS MIN6 cells, which express reduced levels of Cx36, show asynchronous Ca2+ transients; (B) Quantification revealed that the proportion of synchronous cells was higher in WT (black bars) than AS MIN6 cells (open bars), whereas the reverse was true for both asynchronous and silent cells. Data are means + SE of three independent experiments. *p<0.05, **p<0.01 and ***p<0.001 for AS versus WT MIN6 cells. (TIF) [file pone.0041535.s001.tif]

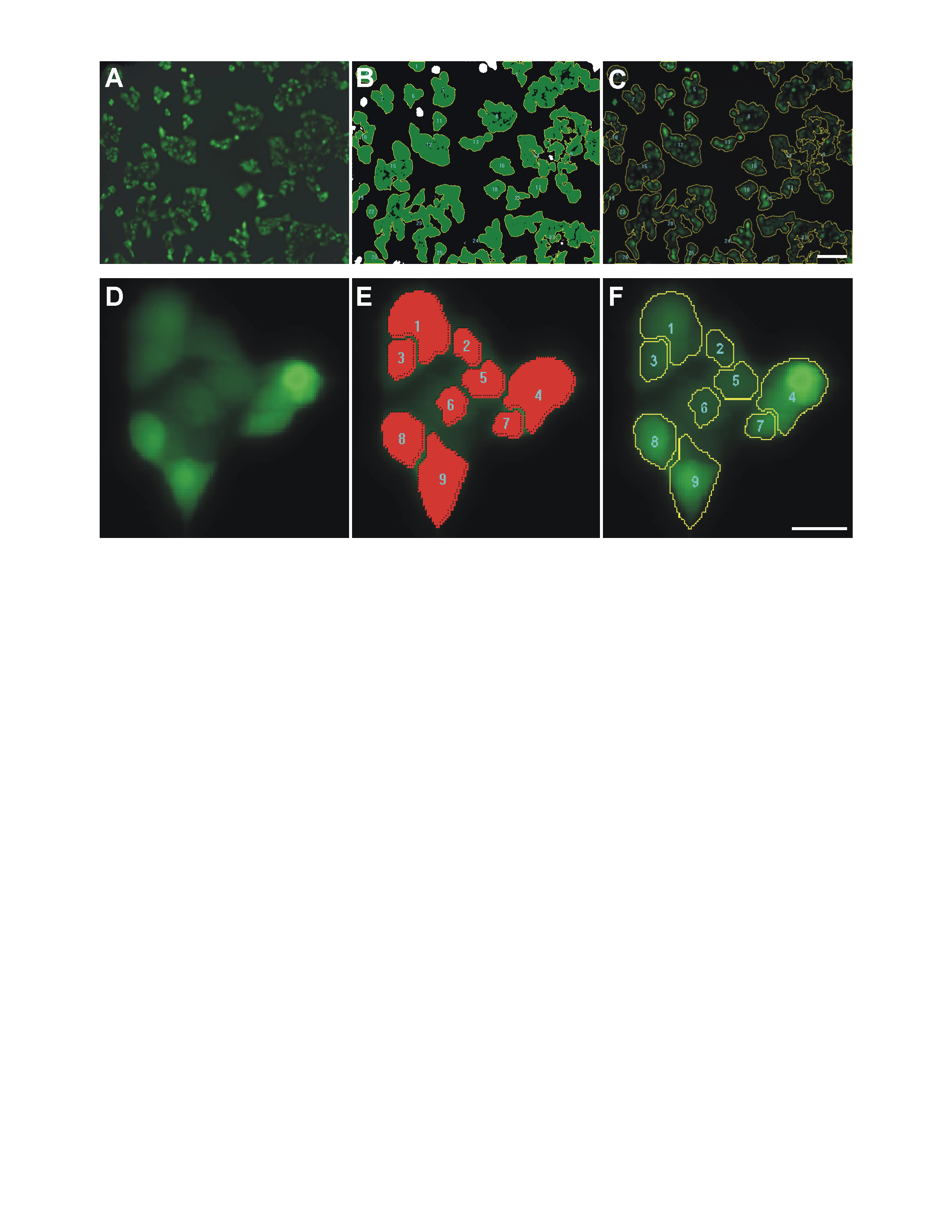

Supplement: Figure S2 — Processing of Fluo-3-loaded MIN6 cells, for evaluation of intercellular Ca2+ synchrony. (A) Low magnification view of clusters of Fluo-3-loaded MIN6 cells, as seen under green fluorescence illumination in the ImageXpress equipment; (B) The software automatically detects clusters comprising more than five cells (green), outlines (yellow line), and identifies them by a number. Clusters of less than five cells are identified separately (white); (C) Clusters are sorted by size, and those containing less than five cells discarded from subsequent calculations; (D) Higher magnification view of one cluster of nine MIN6 cells, featuring a green fluorescence due to Fluo-3 uptake; (E) The same cluster is seen under a rhodamine channel, which detects the regions of highest fluorescence intensity. Deconvolution improves cell detection; (F) A region of five pixel width (yellow line) is automatically defined around each nuclear region to define the ROIs where fluorescence intensity was recorded as a function of time. Bar, 50 µm in A, B and C, and 10 µm in D, E and F. (TIF) [file pone.0041535.s002.tif]

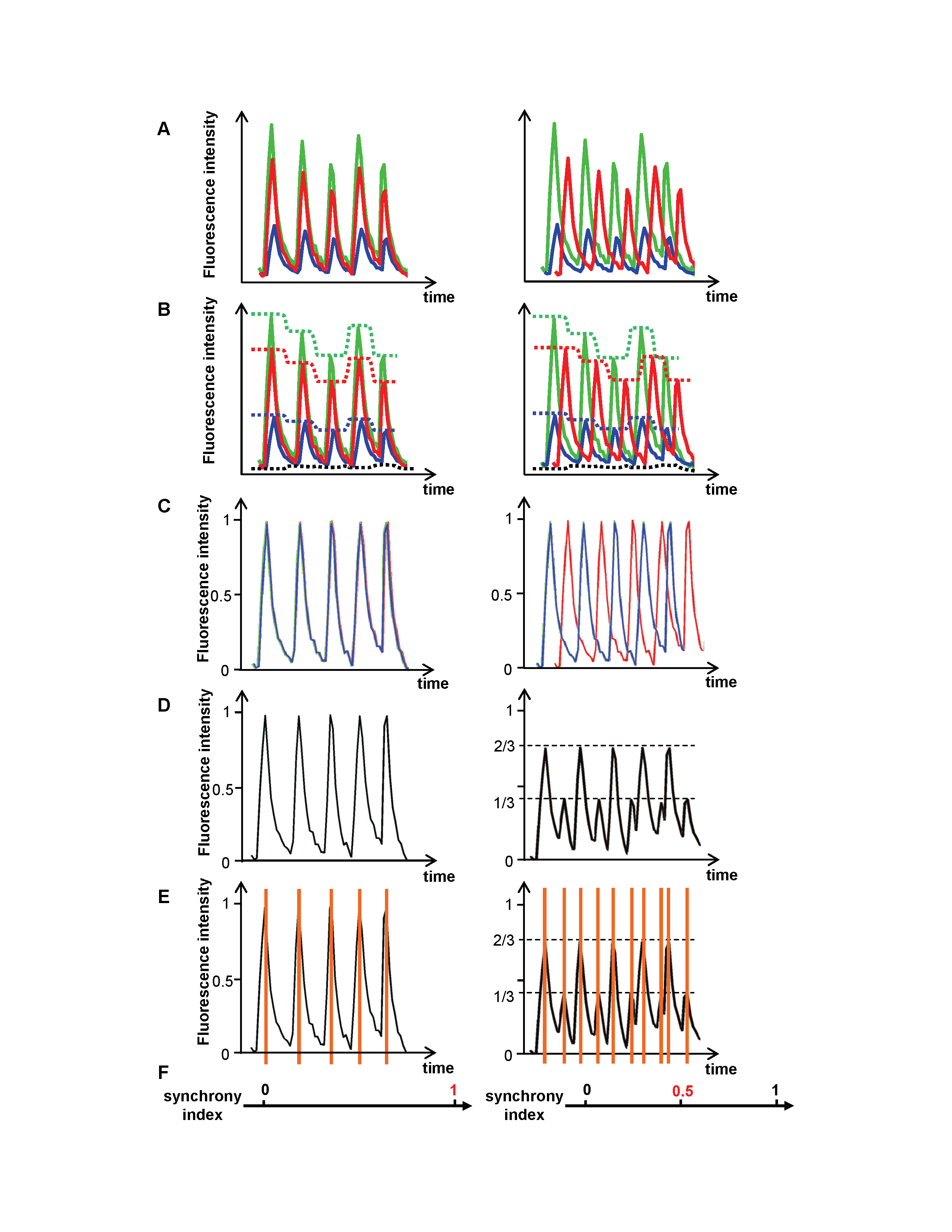

Supplement: Figure S3 — Steps for the automatic evaluation of the “synchrony index”. (A) Records of fluorescence intensity as a function of time are shown for a fully synchronized (left column) and a poorly synchronized cluster (right column). Each colour shows the recording from a different cell (for clarity reason, only three cells were selected for these illustrations); (B) The envelope of each curve was drawn using a morphological opening and closing operation, resulting in a lower local minimum (black dotted line) and an upper local maximum bonds, respectively (coloured dotted lines); (C) The amplitude of each curve was set to 1, to give each cell recording the same weight; (D) An average equalized curve was computed for each cluster; (E) The presence of peaks was detected by computing the derivative of each curve; (F) The average peak values of all the clusters estimated the “synchrony index”, an indicator of the average synchrony of all the MIN6 cells screened in a well. (TIF) [file pone.0041535.s003.tif]

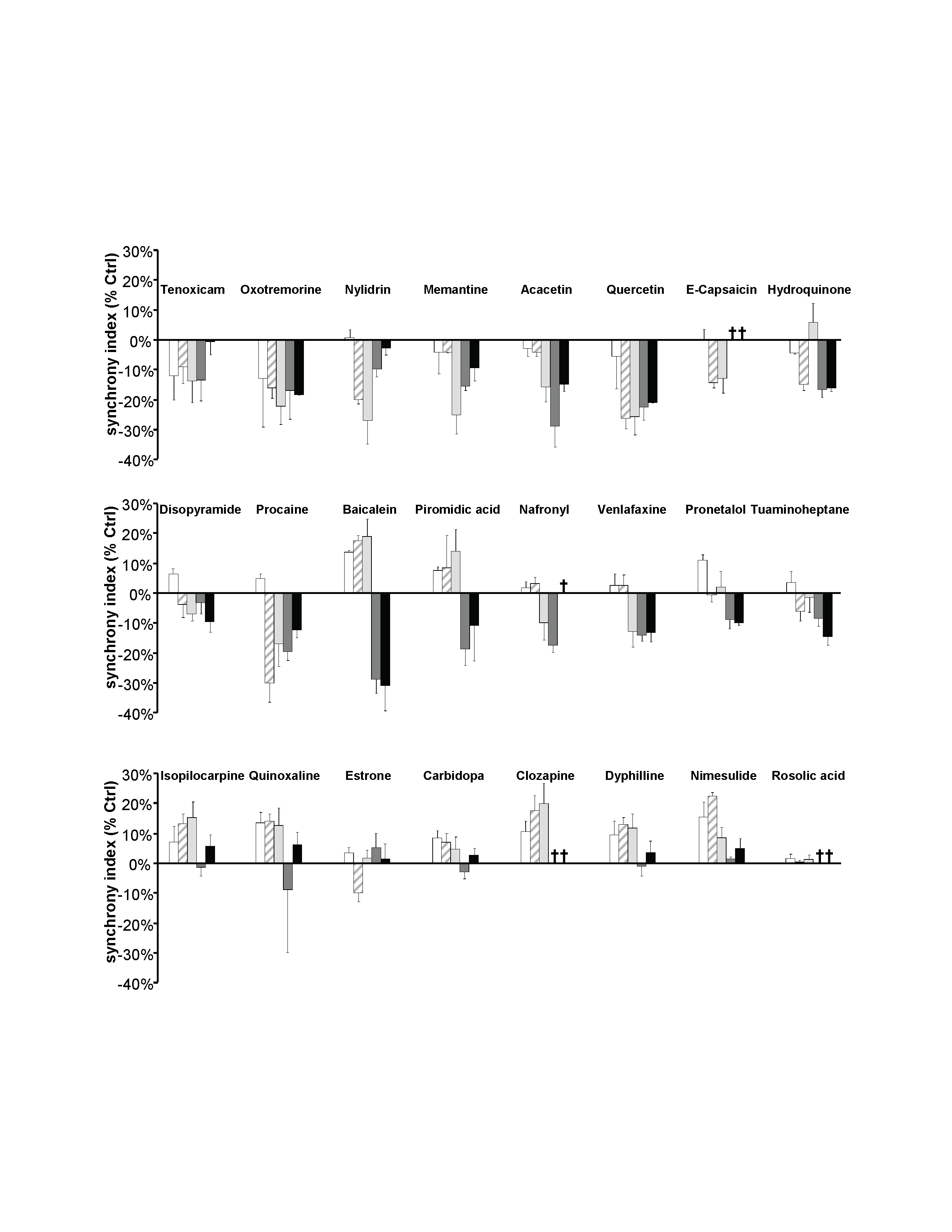

Supplement: Figure S4 — Dose-response relationship of the 24 drugs selected from the primary screening. The effect on synchrony index (relative to that of WT MIN6 cells exposed to DMSO) was plotted as a function of 0.1, 1, 10, 50 and 100 µM concentration of each drug. Data are means ± SE of at least four independent experiments. †indicate toxic concentrations, as evaluated by cell detachment from the support. (TIF) [file pone.0041535.s004.tif]

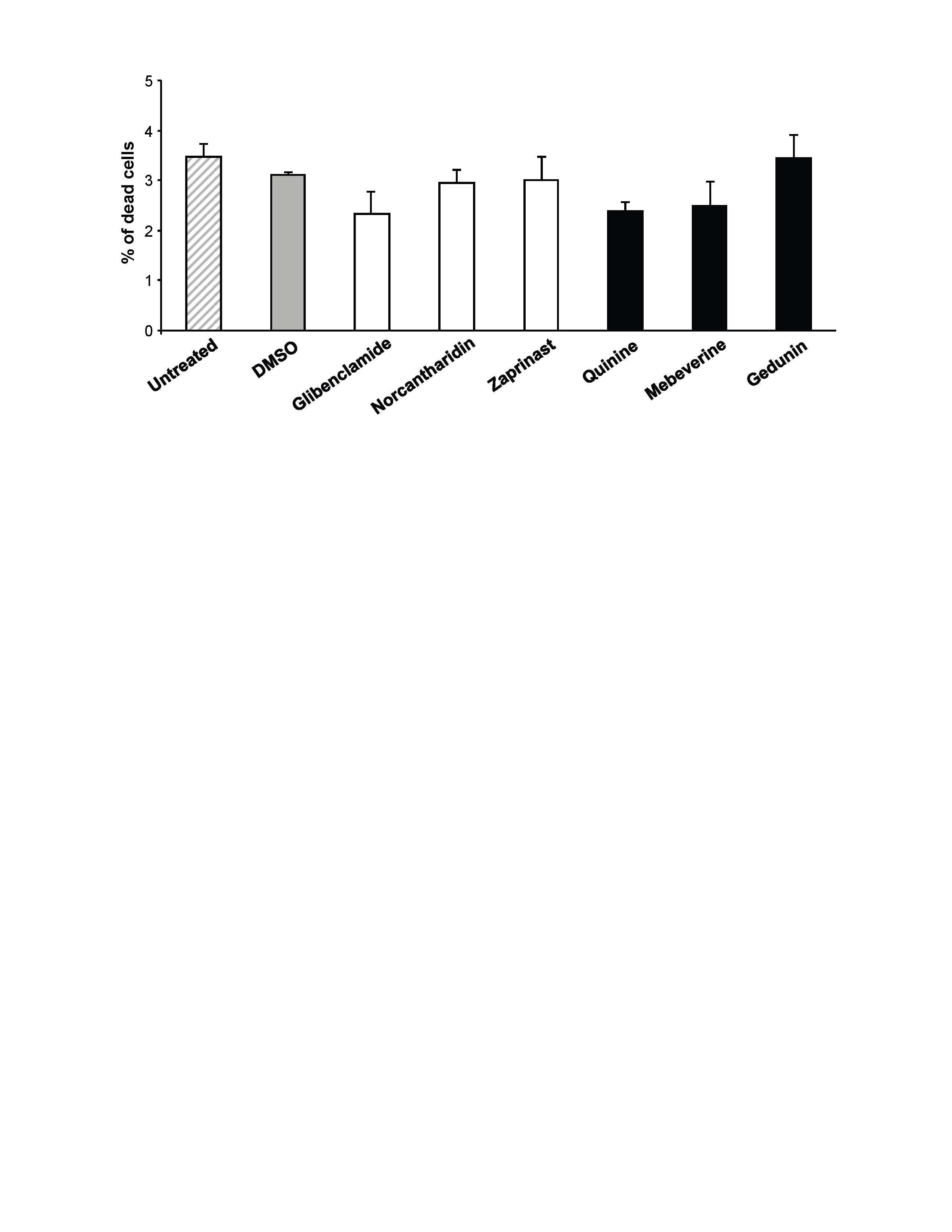

Supplement: Figure S5 — Drugs affecting the synchrony index and Cx36 coupling, and selected from the secondary screening, were not toxic to MIN6 cells. The percentage of dead cells, after a 24 h treatment with 10 µM the different drugs selected from the secondary screening, was comparable to that observed in DMSO-exposed controls. No difference was observed between untreated and DMSO-treated cells. Data are means + SE of three independent experiments. (TIF) [file pone.0041535.s005.tif]

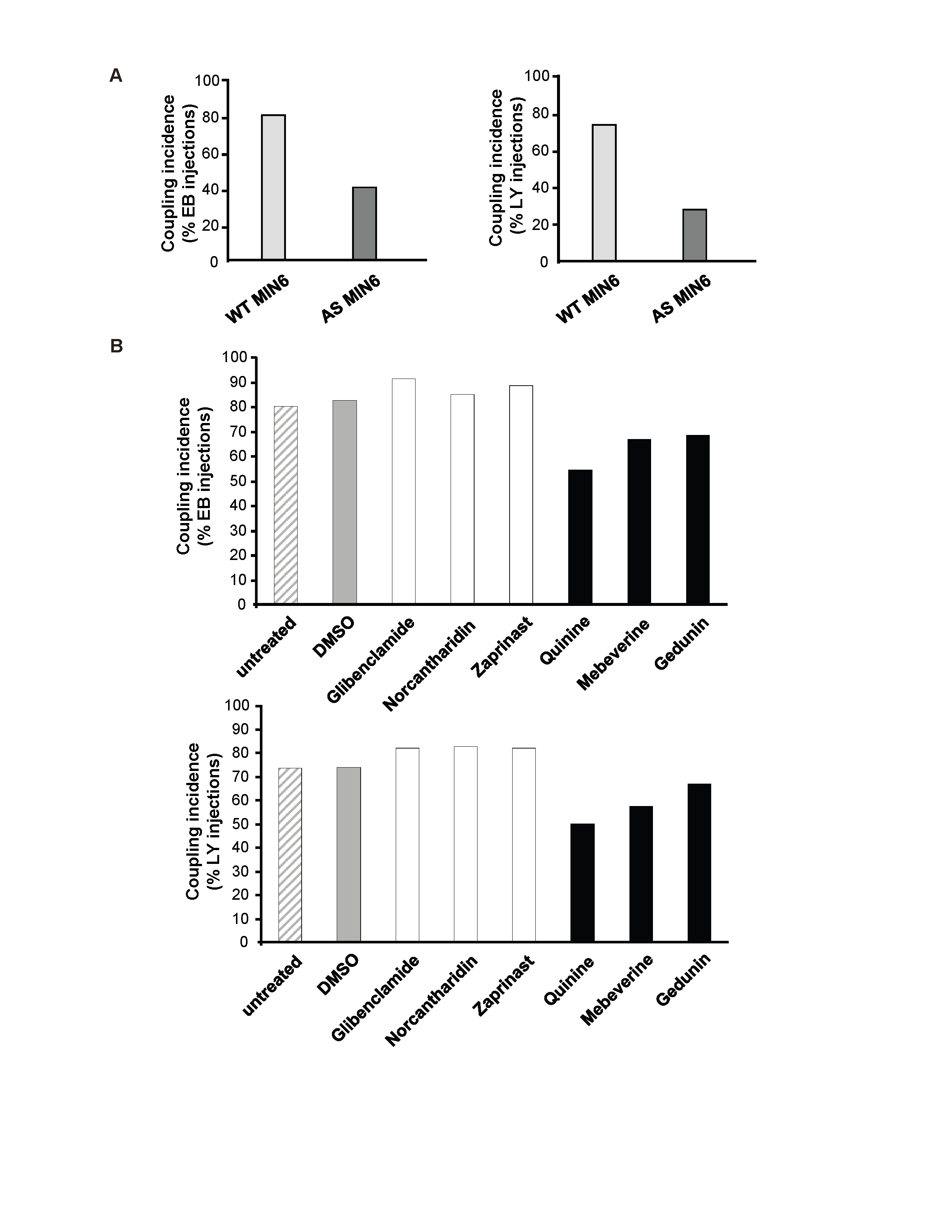

Supplement: Figure S6 — Coupling incidence of MIN6 cells, as investigated by tracer microinjection. (A) The coupling incidence (i.e. the percentage of injections showing cell-to-cell transfer of a tracer) of both Ethidium Bromide (EB, left panel) and Lucifer Yellow (LY, right panel) was markedly reduced in AS MIN6 cells when compared to WT cells. Values are means of four experiments. (B) The coupling incidence of both EB (upper panel) and LY (lower panel) was not markedly affected by the six drugs tested, even though a trend towards an increase and a decrease was observed in the presence of drugs increasing (white bars) and decreasing the synchrony index (black bars), respectively. (TIF) [file pone.0041535.s006.tif]

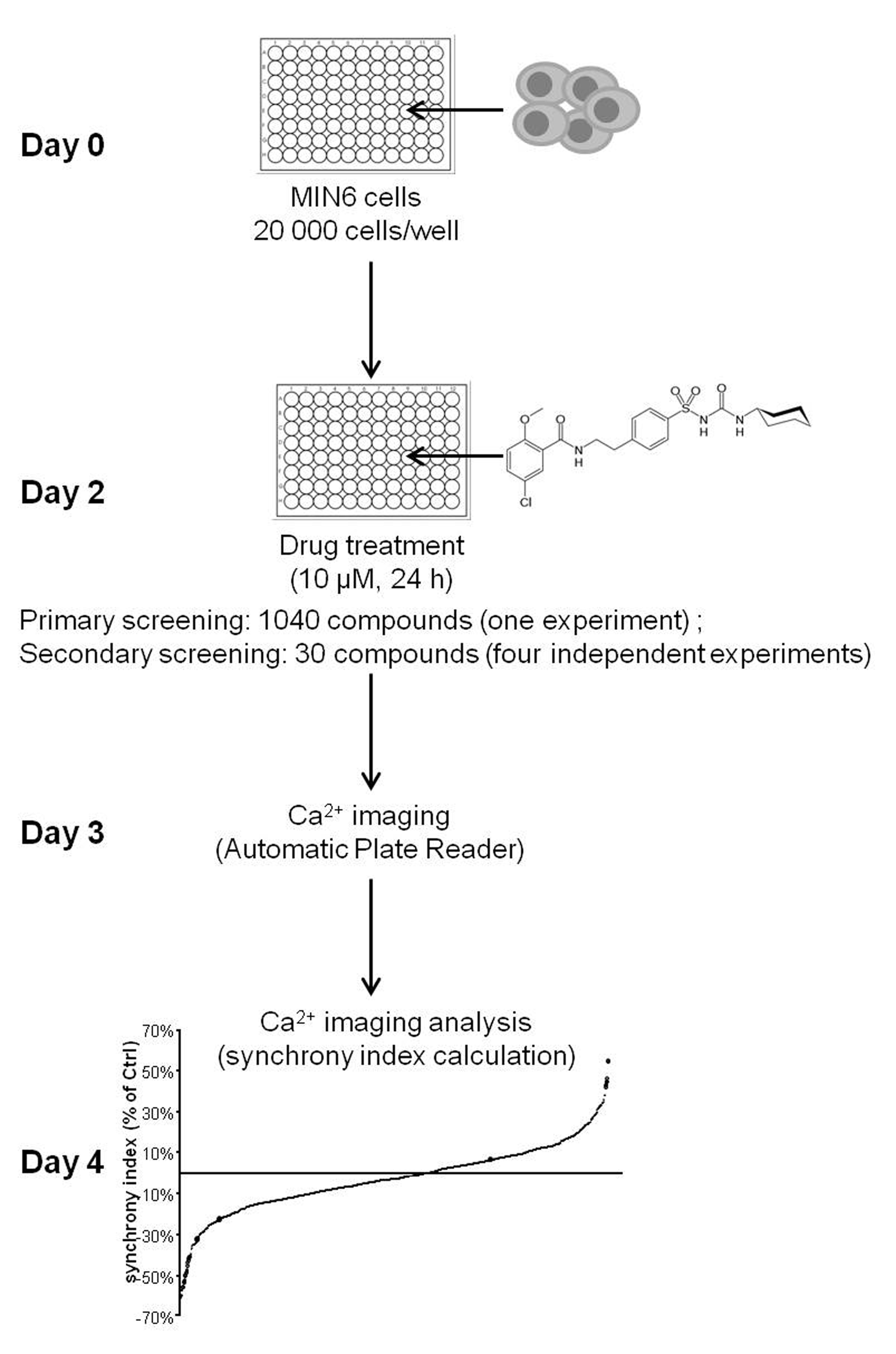

Supplement: Figure S7 — Schematic of the screening protocol. Flowchart of the Ca2+ imaging screening protocol. Day 0: Plating of MIN6 cells in 96-well plate format; Day 2: Compound treatment (10 µM, 24 h); Day 3: Ca2+ imaging; Day 4: Ca2+ imaging analysis. (TIF) [file pone.0041535.s007.tif]
