# Supplementary material for: The Intercellular Synchronization of Ca2+ Oscillations Evaluates Cx36-Dependent Coupling
Source: PLoS One. 2012 Jul 25;7(7):e41535. doi: 10.1371/journal.pone.0041535 (PMC3405138; doi:10.1371/journal.pone.0041535)
Supplement: Materials S1 — Algorithms for the evaluation of intercellular Ca2+ synchronization in large MIN6 cell populations. (DOCX) [file pone.0041535.s009.docx]

**MATERIALS S1**

**Algorithms for the evaluation of intercellular Ca^2+^ synchronization in large MIN6 cell populations**

The ImageXpress software was designed to detect individual cell clusters within each well, evaluate the intercellular synchronization of Ca^2+^ transients within each cluster, and calculate an average “synchrony index” for the entire cell population (Figs. S2 and S3), as per the following steps: i) sequential images of Fluo-3 loaded MIN6 cells (40-50 clusters, each of 6-20 cells, per ROI; one ROI per well) were taken at 488 nm (Fig. S2 *A*); ii) images of individual wells were stacked, and an average image obtained; iii) an image of the same ROI taken at 516 nm was added to improve the uniform imaging of clusters; iv) a threshold was automatically applied to the resulting image, to separate bright objects from dark background, resulting in a proper definition of most clusters (Fig. S2 *B*); v) clusters with an area < 1000 µm^2^ (~ 5 cells) were discarded (Fig. S2 *C*).

Thereafter, each cluster was separately (Fig. S2 *D*) and automatically analyzed, as follows: i) each cluster was imaged under rhodamine fluorescence excitation to detect the position of individual cells, given that nuclei were brighter than cytoplasms (Fig. S2 *E*); ii) the same cluster was seen under green fluorescence excitation to evaluate the total cell area (Fig. S2 *D*); iii) the red image was deconvoluted using the CountNuclei functionality of MetaXpress to improve detection of individual cells. A layer of five pixel width was added around each ROI, defined as a closed polygon, and superposed to the green fluorescence image to outline each cell (Fig. S2 *F*); iv) Ca^2+^ transients were automatically evaluated in each cell, and plotted as average fluorescence intensity as a function of time (Fig. S3 *A*).

Fluorescence intensity values were exported to Excel sheets and analyzed as per the following steps: i) the amplitudes of all individual curves were equalized with respect to the lower and upper bounds, given by a morphological dilation and erosion operation which used a structuring element of 20 time points, respectively (Fig. S3 *B*); ii) the amplitude of each peak was set to 1, to give each cell the same weight in the subsequent calculations (Fig. S3 *C*); iii) curves from each cluster were summed, and an average curve obtained by dividing the sum of curves by their number (Fig. S3 *D*); iv) the resulting curve was analyzed for the presence of significant peaks, by computing the derivative of the curve (Fig. S3 *E*). Peaks were defined as the time this derivative changed significantly from a positive to a negative value; v) the amplitudes of all the peaks evaluated in each cluster were summed, and an average amplitude obtained after division by the number of peaks. The final value, ranging from 0 to 1, was referred to as “synchrony index”, and taken to reflect the synchrony of all cells within a well (Fig. S3 *F*). When cells were fully synchronized, this index was close to 1, whereas when cells were poorly synchronized this index decreased. When all cells were silent, the synchrony index was 0 (Fig. S3 *F*).

A subset of data was also analyzed using the variance of the cluster-averaged fluctuations, normalized by the average of the variances of the cell fluctuations, which is a standard method for evaluating synchrony of various events [[1](#_ENREF_1)]. This analysis provided results similar to those that were obtained by the procedure detailed above (data not shown).

1. Golomb D, Rinzel J (1993) Dynamics of globally coupled inhibitory neurons withheterogeneity. Phys Rev E Stat Phys Plasmas Fluids Relat Interdiscip Topics 48: 4810-4814.
